# Utilization of the Veterans Affairs’ Transgender E-consultation Program by Health Care Providers: Mixed-Methods Study

**DOI:** 10.2196/11695

**Published:** 2019-01-14

**Authors:** John R Blosnich, Keri L Rodriguez, Kristina L Hruska, Dio Kavalieratos, Adam J Gordon, Alexis Matza, Susan M Mejia, Jillian C Shipherd, Michael R Kauth

**Affiliations:** 1 Center for Health Equity Research and Promotion Department of Veterans Affairs Pittsburgh Healthcare System Pittsburgh, PA United States; 2 Division of General Internal Medicine, Department of Medicine School of Medicine University of Pittsburgh Pittsburgh, PA United States; 3 Informatics, Decision-Enhancement, and Analytic Sciences Center Department of Veterans Affairs Salt Lake City Health Care System Salt Lake City, UT United States; 4 Department of Internal Medicine School of Medicine University of Utah Salt Lake City, UT United States; 5 Lesbian, Gay, Bisexual, and Transgender Health Program Office of Patient Care Services Veterans Health Administration Washington, DC United States; 6 South Central Mental Illness Research, Education and Clinical Center Michael E DeBakey Veterans Affairs Medical Center Houston, TX United States; 7 National Center for Posttraumatic Stress Disorder Department of Veterans Affairs Boston Healthcare System Boston, MA United States; 8 Boston University School of Medicine Boston, MA United States; 9 Department of Psychiatry Baylor College of Medicine Houston, TX United States

**Keywords:** teleconsultation, telemedicine, transgender persons, veterans, veteran health

## Abstract

**Background:**

In 2015, the Department of Veterans Affairs (VA) nationally implemented a transgender e-consultation (e-consult) program with expert clinical guidance for providers.

**Objective:**

This mixed-methods project aimed to describe providers’ program experiences, reasons for nonuse of the program, and ways to improve the program use.

**Methods:**

From January to May 2017, 15 urban and rural VA providers who submitted at least one e-consult in the last year participated in semistructured interviews about their program experiences, which were analyzed using content analysis. From November to December 2017, 53 providers who encountered transgender patients but did not utilize the program participated in a brief online survey on the reasons for nonuse of the program and the facilitators encouraging use.

**Results:**

Qualitative analysis showed that providers learned of the program through email; colleagues; the electronic health record (EHR) system; and participation in the VA Lesbian, Gay, Bisexual, and Transgender committees or educational trainings. Providers used the program to establish care plans, hormone therapy recommendations, sexual and reproductive health education, surgical treatment education, patient-provider communication guidance, and second opinions. The facilitators of program use included understandable recommendations, ease of use through the EHR system, and status as the only transgender resource for rural providers. Barriers to use included time constraints, communication-related problems with the e-consult, impractical recommendations for underresourced sites, and misunderstanding of the e-consult purpose. Suggestions for improvement included addition of concise or sectioned responses, expansion of program awareness among providers or patients, designation of a follow-up contact person, and increase in provider education about transgender veterans and related care. Quantitative analysis showed that the common reasons for nonuse of the program were no knowledge of the program (54%), no need of the program (32%), and receipt of help from a colleague outside of e-consult (24%). Common suggestions to improve the program use in quantitative analyses included provision of more information about where to find e-consult in the chart, guidance on talking with patients about the program, and e-mail announcements to improve provider awareness of the program. Post hoc exploratory analyses showed no differences between urban and rural providers.

**Conclusions:**

The VA transgender e-consult program is useful for providers, but there are several barriers to implementing recommendations, some of which are especially challenging for rural providers. Addressing the identified barriers and enhancing the facilitators may improve program use and quality care for transgender veterans.

## Introduction

Transgender is an umbrella term that encompasses individuals with gender identities (ie, the core sense of self as male, female, both, or neither) that conflict with societal prescriptions of masculinity and femininity associated with sex assigned at birth. Transgender individuals experience numerous health disparities [1] including health risk (eg, victimization [2]), health outcomes (eg, depression and HIV [3]), and barriers to health care access [4], all of which can create complex care needs. In addition, they often require medical care specific to their gender affirmation (ie, gender experience reflecting their gender identity) including mental health services; evaluations for hormone therapy; prescription and monitoring of hormones; gender-affirming surgery and postoperative care; and specialty services such as urology, endocrinology, and speech therapy [5]. In addition to specific services, there are unique needs related to documentation, particularly sex assigned at birth, which health systems often link to other important medical services including laboratory test values, medication dosages, and critical health screens such as breast or prostate examinations. Although awareness of care for transgender patients is growing, health system-level efforts to improve transgender patients’ care are limited. There is a paucity of formative research on how providers can collaborate with specialists to obtain expert clinical guidance on transgender-specific care for their transgender patients [6].

Achieving equity in health and health care, irrespective of gender, is a specific objective for the Department of Veterans Affairs (VA) [7]. The VA has a higher rate of transgender individuals than the United States (US) population [8], and the transgender veteran population has increased annually in the VA over the last decade [9]. Recognizing the need to serve transgender veterans, in 2011, the VA issued a national policy on health care for transgender veterans. To increase the VA’s capacity to provide high-quality care to transgender veterans, the Lesbian, Gay, Bisexual, and Transgender (LGBT) Health Program in the VA Office of Patient Care Services developed a transgender e-consultation (e-consult) program to provide expert clinical guidance for VA health care providers; the program was implemented nationally in April 2015 and was described previously [10].

Generally, e-consults provide a technology-based platform through which teams of providers with expertise in a specific medical condition or process of care can assist providers in medical centers where experts may not exist locally [11]. The VA program allows transgender veterans to receive health care from their local providers, with remote support from experts in transgender health care, thus reducing or eliminating the need for veterans to travel to tertiary VA sites for care. The program includes interdisciplinary teams with expertise in transgender health care at three sites: Minneapolis, Tucson, and Loma Linda. Each expert team comprises at least 4 members (eg, primary care physician, psychologist, social worker, nurse, endocrinologist, and pharmacist). Consultations typically address 4 main topics: mental health evaluation and hormone readiness (eg, questions about diagnosis of gender dysphoria and assessment of readiness for gender-affirming surgery and hormone therapy); psychotherapy (eg, gender counseling and gender-informed treatment for comorbid conditions); primary medical questions (eg, addressing expected outcomes, assessing medical risks, and recommended monitoring of gender affirmation pharmacologically and medically); and prescription of hormone therapy (eg, how to safely prescribe hormone therapy for gender affirmation).

Since the e-consult is a relatively new resource for VA providers, there is a critical need for formative evaluation that can guide future implementation research [12] to, for example, examine how this program serves system needs and explore how the program can be improved through minor adjustments. Ultimately, understanding the processes and outcomes of the VA transgender e-consult program is critical for measuring its impact on enhancing care for transgender veterans. This project aimed to describe providers’ program experiences through qualitative analysis, determine the reasons for nonuse of the program, and identify methods for improving program use through a quantitative survey.

## Methods

### Ethical Considerations

All qualitative activities were conducted as part of a quality-improvement initiative by the directors of VA’s LGBT Health Program in the Office of Patient Care Services; therefore, no subject approval or informed consent to participate was required. The quantitative analyses were approved by the Institutional Review Board of the VA Pittsburgh Healthcare System.

### Qualitative Methods

We received the LGBT Health Program’s list of unique VA providers who used the VA transgender e-consult program, from the program’s national launch in April 2015 through December 2016. We categorized the provider roster by urban and rural sites based on urban-rural definitions of the VA Office of Rural Health [13]. Subsequently, we randomly selected 32 users from urban sites and 34 users from rural sites. In January 2017, the selected providers received a recruitment email from the LGBT Health Program that explained the project goals and invited them to participate in a 20- to 30-minute semistructured phone interview to discuss their experiences with the transgender e-consult program. Approximately 1 week later, a project team member sent a follow-up email to the pertinent providers and reached out via direct phone calls. These efforts resulted in interviews with 3 urban and 6 rural providers. In May 2017, this process was repeated for providers who had not yet participated in the interviews, resulting in additional interviews with 1 urban and 5 rural providers (a total of 4 urban and 11 rural provider interviews).

With guidance from personnel who direct and administer the e-consult program, we developed a semistructured interview guide based on the most-germane needs identified. The initial set of guiding questions were as follows: How did providers learn about the availability of e-consultation? How useful was the e-consultation response? How did the treatment plan change as a result of the consultation? Were there provider-perceived changes in patient outcomes as a result of the consultation? What were the challenges or limitations of the e-consultation program? From the guiding questions, the project team developed and finalized an interview guide with open-ended questions and additional probes to direct in-depth inquiry. From January to May 2017, a trained interviewer conducted interviews, which were audio-recorded and transcribed for analysis.

Microsoft Word 2016 (Microsoft Corp., Redmond, WA) was used to transcribe the audio recordings verbatim to identify categories across interviews. The interviews were analyzed using conventional qualitative content analysis [14]. Using inductive analysis, the narrative text was reviewed using open coding. The coder read the interview transcripts multiple times to develop and refine categories in order to describe health care providers’ experiences using the e-consult program. The lists of categories were sorted and grouped according to similar content for various aspects of providers’ experiences in using the program under higher-order headings, with reduction of the data by content areas.

We performed quality control and bias mitigation in several ways. First, an experienced qualitative researcher reviewed the codebook prior to completion of the coding process. Second, the same researcher reviewed 20% (4 transcripts) of the samples of coded transcripts to ensure transparency and comprehensibility in the application of codes. The results were subsequently reviewed and discussed by three of the authors before their finalization.

### Quantitative Methods

For the survey portion of our project, VA’s administrative data were extracted from April 1, 2015, through April 1, 2017, for all inpatient and outpatient visits in which a transgender-related International Classification of Diseases (ICD)-9 or ICD-10 diagnosis code was noted for the visit (eg, gender-identity disorder, transsexualism, and personal history of sex reassignment). For each visit, the names of all unique providers who noted the diagnosis code were extracted to create a census of providers who encountered a transgender patient during the study period. The list of providers who had used the transgender e-consult program during the study period was cross-referenced with the total list of providers who encountered a transgender patient, and the providers who used the e-consult were removed. The remaining providers represented the eligible sample of providers who had encountered transgender patients during the study period, but did not utilize the e-consult program (n=14,502). Of the eligible providers, a random sample of 300 providers was selected, regardless of the provider type (eg, social worker or primary care physician). Email addresses of all providers were manually collected from the VA’s global contact list, and addresses of 279 providers (of 300, 93%) were available, which comprised the analytical sample for this project. Providers received a recruitment email on November 13, 2017, which explained the purpose of the research project and provided a link to a brief 9-item survey on VA’s internal network via REDCap (Vanderbilt University, Nashville, TN). Two weeks later (November 27), a second recruitment email was sent to all providers who had not completed the survey.

In the brief online survey, VA providers were asked to identify their discipline from social work, psychology, psychiatry, primary care, primary care nursing, endocrinology, speech therapy, nursing, and other. Providers indicating “other” were asked to elaborate on their answer. Before beginning the project with the VA, providers were asked if they ever received any training on providing health care to transgender patients; response options were yes, >1 hour; yes, >1 to <3 hours; yes, ≥3 hours; and no. Using the same response options, participants were asked if they ever received training when working for the VA. In addition, providers indicated the number of transgender patients they had cared for in the last 12 months. Finally, providers were asked, “Based on your own perception of the VA facility in which you do most of your work, how would you classify your VA facility?” The response options were urban, rural, or highly rural.

Three questions focused on the transgender e-consult program. First, providers indicated if they had ever used the program. For individuals who indicated “yes,” they were thanked for their participation, and the survey ended. Individuals who indicated that they had not used the e-consult program received two additional questions. The first queried the providers about the issues that prevented them from using the program, and the response options included the following: I did not know about it, I do not know how to access it, I have not needed to use it yet, I am comfortable with my level of knowledge with transgender care, my patient(s) told me they did not want me to use it, I have a colleague I can call on for help, template takes too long to complete, and other. The second item queried providers about the factors that would encourage them to use the program. Response options included the following: provision of more information about where to find e-consult in the chart, allotment of more time to complete an e-consult, encountering more transgender patients, VA-wide email announcements about the program, guidance on how to talk with patients about the program, and other. For both items, providers could choose more than one option, and persons indicating “other” were asked to elaborate on their answer.

Univariate statistics were used to describe the sample. Chi-square tests of independence and *t*-tests were used to examine categorical and mean differences, respectively. All quantitative analyses were conducted using Stata or SE, version 14.2 (StataCorp, College Station, TX). Values of *P*<.05 were considered statistically significant.

## Results

### Qualitative Evaluation

#### Participant Characteristics

Of the 15 interview participants included, most were primary care providers (12/15), practiced at VA for >10 years (7/15), categorized their current VA facility as rural (9/15), had no transgender-specific training (9/15), encountered 1 transgender patient in the last year (6/15), and reported that the program was 90%-100% useful for their specific needs (7/15) (Table 1). In addition, 27 of the total consultations were e-consults, and the majority (9/15) of the providers used the program at least once.

Fourteen VA health care providers participated in a telephone-based interview, of which 1 participant sent written responses via email because they could not schedule an interview. The longest interview lasted for 30 minutes 19 seconds, and the shortest was of 7 minutes 36 seconds; the mean interview time was 18 minutes 9 seconds. Six major areas were identified: how providers learned about the e-consult program, reasons for using the program, results of using the program, facilitators for using the program, barriers to using the program, and suggestions for improving the program. Table 2 includes exemplar quotes for each of the following categories identified during qualitative analyses.

#### Ways Providers Learned of the E-Consult Program

Providers commonly received information about the program through an email from the VA’s LGBT Health Program. Local providers or colleagues such as pharmacists, mental health providers (eg, psychologists), and gynecologists also educated interviewees before the e-consult as well as when another provider asked for assistance or advice through “word of mouth.” In addition, providers learned about the program by seeing “Transgender E-Consultation” as an option in the VA electronic health record (EHR). Some providers saw this option when entering an e-consult request in a different specialty area, whereas others saw it while going through orders. Other providers learned about the program through participation in local VA LGBT-specific activities or educational lectures for providers about transgender care.

#### Reasons for Using the E-consult Program

Providers used the program to create individual patient-care plans, particularly providers with “extremely limited” transgender care experience. Some examples focused on gender-affirming medical care, including determining whether the patient was a good candidate for medical transition and the type of monitoring necessary during the transition process. Others sought recommendations about hormone therapies. Although there were “general medication questions,” most asked specific questions about appropriate medication doses and adjustments to optimize therapeutic outcomes and address safety concerns (eg, potential adverse effects). Furthermore, providers used the consult for education about sexual and reproductive health to help make care decisions such as those regarding standard preventative health-screening tests (eg, mammograms for transgender women) and gynecology for transgender men. Others desired education about available surgical treatment options (eg, breast augmentation and bilateral mastectomy). Moreover, providers sought guidance for communication with transgender patients on, for example, information about hormone therapy they should provide their patients and the responses they should expect. E-consults also provided a second opinion when providers were uncertain about a health care decision, especially in complex cases or cases involving high risk. The consults were found to be especially helpful when they confirmed what the provider already informed the patient or helped bolster provider decisions that were made against patient preferences.

#### Results of Using the E-consult Program

Providers sometimes modified care plans for their transgender patients after obtaining an e-consult response, including a formal diagnosis. Modifications included addition of recommended gender dysphoria evaluations, health screenings, medication monitoring, and meeting standard time frames for follow-up care. Most treatment plan changes were related to dosage of hormone therapy and addition of specialist referrals. Providers who used the program became more comfortable and confident in their assessments and in relaying testing and treatment decisions for transgender patients. Some providers felt confident in their judgment before the e-consult, but a second opinion confirmed what they discussed and decided with the patient. E-consults helped improve patient-provider relationships by placing those interactions and care provision in line with the gender (ie, body, identity, and expression) of their patients. In addition, the e-consults provided patients added motivation to follow recommendations because experienced providers were involved. Moreover, communication during clinic interactions improved because the e-consult request requires providers to answer a number of questions about the patient, which encouraged them to collect additional relevant information from the patient during their visit. Discussing recommendations with their patients allowed them to engage in informed, shared decision making about their care.

**Table 1:** Characteristics of the 15 Department of Veterans Affairs (VA) providers from qualitative interviews.

Characteristics		Providers, n (%)
**Type of VA health care provider**		
	Primary care	12 (80)
	Mental health	3 (20)
**Transgender-specific training (in VA and nonVA settings)**		
	No	9 (60)
	Yes	6 (40)
**Years in practice at VA**		
	0-3 years	3 (20)
	>3-5 years	2 (13)
	>5-7 years	1 (7)
	>7-10 years	2 (13)
	>10 years	7 (47)
**Number of transgender patients in the last year**		
	1	6 (40)
	2	3 (20)
	3	3 (20)
	4	2 (13)
	>5	1 (7)
**Number of e-consults performed**		
	1	9 (60)
	2	4 (27)
	3	1 (7)
	4	0 (0)
	5	0 (0)
	6	0 (0)
	7	1 (7)
**Usefulness of the e-consult program**		
	>90%-100%	7 (47)
	>80%-90%	4 (27)
	>70%-80%	1 (7)
	>60%-70%	1 (7)
	>50%-60%	0 (0)
	≤50%	1 (7)
	No answer	1 (7)
**Location of facility (participant self-report)**		
	Rural	9 (60)
	Urban	3 (20)
	Suburban or mixed	2 (13)
	No answer or not asked	1 (7)
**Location of facility (defined by the Office of Rural Health)**		
	Rural	11 (73)
	Urban	4 (27)

**Table 2:** Qualitative findings among providers using the Department of Veterans Affairs (VA) transgender e-consult program.

Categories		Quotation examples
**Topic 1: Ways providers learned of the program**		
	Email notification	“There was an email with a flyer.”
	Informed by other staff members	“My pharmacist or my mental health provider at the clinic showed me how to access that consult.”
	Via the EHR^a^ system	“Through the VA Consult Program, ...through the orders, you can go to the e-consult request and order sections in the records and there’s ‘Transgender E-Consults’ you can click on.”
	Participation in VA LGBT^b^ activities and educational trainings	“Through one of those lectures that we got about transgender care.”
**Topic 2: Reasons for using the program**		
	Establish a care plan	“My experience with the gender reassignment is extremely limited.”
	Recommendations about hormone-replacement therapies	“I was having too much testosterone and I needed to...see if I was doing something not quite right. They gave me some tips like, ‘Yes, we want this level for the testosterone in order for...transition’.”
	Education about sexual and reproductive health care	“I wasn’t sure how to screen her because I didn’t know...how often to get her a mammography.”
	Education about surgical treatment options	“What options we might have available for surgical interventions.”
	Guidance regarding patient-provider communication	“I typically will ask about what types of responses should I expect...What should I tell patients about what type of breast size increase they may expect.”
	Second opinion	“This fellow is a senior and he’s got multiple medical problems and he’s on anticoagulants, and lots of cardiac issues, and hormones would be a really bad idea. (I used the consult to) just evaluate him and see if he’s a candidate.”
**Topic 3: Results of using the program**		
	Care-plan modifications	“It was helpful for outlining starting medicines and then following the intervals for following those with laboratories.”
	Increased provider comfort with and confidence in transgender care provision	“They actually had a template to walk someone through it. And it was exceptional. Because, while I have worked with this population before, I really, at that point, wasn’t comfortable starting from scratch in the assessment.”
	Improved patient-provider relationships	“I thought (the e-consult) did ask questions that kind of allowed me to ask the patient more questions. So, it kind of delved a bit, so that if you are new to it...”
	Continued use of the e-consult program	“I’ve only offered it to the one patient, but on multiple occasions.”
**Topic 4: Facilitators for using the program**		
	Responses contain understandable and informative recommendations	“I do exactly what they say, and if it doesn’t work they say ‘This is the next step you should take.’ They’ll say, ‘Go ahead and give us a call back.’”
	Accessibility and ease of program use by providers through the EHR	“It was just on our primary (EHR) page, so it was really easy to find... When we put in for consults, it’s on the very main screen.”
	Quick response to e-consult request	“They answered my inquiry right away.”
	Provider knowledge of the program’s existence	“Knowing it was out there.”
	Only available transgender-specific resource for providers at rural sites	“I don’t have much of a choice. The only resource we have is to use the e-consult.”
**Topic 5: Barriers to using the program**		
	Time-consuming process for the provider to submit an e-consult and read through recommendations	“They pull in parts of notes from the mental health evaluation, from my evaluation, from all sorts of things. So, the note is not clear when it comes back... They probably have a dozen pages where they (explain) hormone levels, when they should be checked... It’s meant to prevent re-consult, which is frustrating. It’s very cumbersome...all that stuff just automatically gets tacked onto the bottom of the consult.”
	Previous e-consult response did not answer the question asked	“One of the responses I received in the e-consult was that I should become an advocate and get more programs. And, honestly, that answer left me feeling demoralized. It was just unrealistic given my workload.”
	Communication-related problems with e-consult	“It’s not really clear from the consult, they want you to submit another e-consult...sometimes you may, six months later, have a question...that may require the submission of a new e-consult (rather than just adding a comment to the existing consult).”
	Impractical e-consult recommendations	“The recommendation (to) see the patient back in a couple months and the follow-up intervals they recommended, to be honest I can’t accommodate.”
	Provider misunderstanding of e-consult purpose	“I just don’t know why they didn’t take the case and take over.”
	Lack of provider understanding of transgender patients and their specific health care needs	“I had been kind of thinking of her as a man in my head... I had been seeing her for about a year before I had even thought about this... I think it was just a lack of awareness.”
**Topic 6: Suggestions to improve the program**		
	Adding concise and sectioned e-consult responses	“Appropriate hormone levels, intervals for checking labs, any changes in preventative care, if those were in separate sections. Because now...it’s just kind of merged into one on-going paragraph.”
	Expanding provider and patient awareness about the program	“Perhaps market it more...I’m pretty sure not a lot of people know it exists.”
	Designating a contact for follow-up after the e-consult	“To have a contact person who I could have more of a dialogue with. There were multiple people contributing to the e-consult, but no one contact person that I could ask logistical questions.”
	Increasing provider education about transgender veterans and transgender-specific health care	“Maybe a little more education on the consult...the consult education was provided after they...look at things and then they were like, ‘Oh, well, this was what you needed to do.’ But if, in the consult itself, you list all of the pre-requisites that are necessary (for transitioning).”
	Clarification for providers on e-consult operational processes and timelines	“Some idea of the turnaround process would have been helpful.”

^a^EHR: electronic health record.

^b^LGBT: lesbian, gay, bisexual, and transgender.

#### Facilitators for Using the E-consult Program

E-consult responses contained “timely and up-to date-information” that was useful and easily understood because it did not contain jargon. They appreciated the fact that the e-consult was reviewed by an expert multidisciplinary team in transgender care and saw them as “coaches.” The recommendations reportedly provided a clear “road map” for where to start and how to proceed with care. The program was said to be “easy to access” and use through the VA EHR system, which also made it “easier for continuity of care” because information such as test and treatment results can be added, accessed together, reviewed, and incorporated into a patient’s care plan.

Providers who used e-consults received responses in a “timely manner,” ranging from 2 days to 2 weeks, depending on the question, which was much shorter than the time for other types of consultations they requested in the past. The program made providers aware of other existing health care services and resources (eg, sexual and reproductive health care services). Some rural providers said they used the e-consult program because they believed it was their only available resource for patient-specific transgender health. In particular, providers at rural sites said there were no on-site providers or resources they could consult about care for transgender patients.

#### Barriers to Using the E-consult Program

The providers reported that entering the required information to submit an e-consult and reading the responses could be time consuming. For example, providers had to enter extensive patient information, some of which was not directly relevant to their question (eg, “Mental health things like suicide or homicide, interpersonal violence”). Some providers thought the template contained repetitive questions. Others said the responses were too long (eg, “If you printed it off, it would probably be like 20 pages long”) and not user friendly, including templated information and irrelevant information that they would not use. One provider was frustrated when the response did not address her question and suggested unrealistic and broad changes in her practice.

Communication-related issues were noted with receipt of the response and follow-up questions, some of which related to the technology central to e-consults. For example, in a situation with multiple e-consults for different questions about the same patient over time, it was unclear whether providers should add a new e-consult or add comments to their previous e-consult. Providers were also unable to follow recommendations because they found them impractical or unfeasible (eg, too busy due to their existing workload). Some providers at rural facilities could not follow recommendations because they lacked the necessary local resources, and the e-consult did not help them access the required resources. A few providers misunderstood the e-consult purpose, which led them to misuse the e-consult and question why their request was not met. For example, some providers thought a referral request would result in other providers taking over their patient’s care. Some providers did not use e-consult due to a lack of understanding about transgender patients, including dimensions of gender (ie, body, identity, and expression) and transgender-specific health care needs.

#### Suggestions for Improving the E-consult Program

Providers suggested streamlining responses into sections to make them easier to read and understand. Redundant or templated sections could be moved to a common share point on the VA intranet. Additionally, providers suggested increasing provider and patient awareness of the program; for example, “Getting information out that, if the veteran has concerns, they can go to their primary care clinics and get the process started, so they don’t have to go outside the VA.” Providers also suggested designating a point-person after an e-consult is completed, so that providers can follow-up and discuss the issue, and not just receive a one-sided, one-time response. Further, they suggested provision of additional provider education about transgender-specific health care needs through the e-consult and the VA Specialty Care Access Network-Extension for Community Healthcare Outcomes program. Creation of new programs would help educate providers about transgender veterans, including dimensions of gender and transgender-specific health care. Providers suggested that additional information and clarification be given to providers about the e-consult program’s operational processes and timeline for e-consults, including the estimated time required to enter an e-consult request and average wait time for a response.

**Table 3:** Comparison between urban and rural providers in 53 provider surveys.

Parameter		Urban (n=35)	Rural (n=18)	*P* value
**Discipline, n (%)**				
	Social work	6 (17.6)	2 (11.1)	.82
	Psychology	7 (20.6)	3 (16.7)	—^a^
	Psychiatry	4 (11.8)	3 (16.7)	—
	Primary care	5 (14.7)	3 (16.7)	—
	Nursing	5 (14.7)	4 (22.2)	—
	Other	7(20.6)	3 (16.7)	—
**Training in transgender health prior to working with the VA^b^, n (%)**				
	Yes, 1 hour	5 (14.7)	1 (5.6)	.72
	Yes, >1 to <3 hours	8 (23.5)	3 (16.7)	—
	Yes, >3 hours	4 (11.8)	3 (16.7)	—
	None	17 (50.0)	11 (61.1)	—
**Training in transgender health while working with the VA, n (%)**				
	Yes	19 (57.6)	7 (38.9)	.20
	No	14 (42.4)	11 (61.1)	—
**Prior use of transgender e-consult, n (%)**				
	Yes	2 (5.9)	3 (16.7)	.33
	No	32 (94.1)	15 (83.3)	—
**Reasons for nonuse of the e-consult, n (%)**				
	Did not know about it	17 (50.0)	12 (66.7)	.25
	Do not know how to access it	7 (20.6)	5 (27.8)	.56
	Have not needed to use it yet	12 (35.3)	5 (27.8)	.58
	Comfortable with knowledge of transgender care	6 (17.6)	1 (5.6)	.40
	Patient(s) told me they did not want me to use it	0	0	—
	I have a colleague I can call on for help	11 (32.3)	2 (11.1)	.18
	Template takes too long to complete	0	0	—
**Suggestions to encourage use of the e-consult, n (%)**				
	More information about where to find e-consult in the chart	18 (52.9)	11 (61.1)	.57
	Have more time to complete an e-consult	6 (17.6)	4 (22.2)	.72
	Seeing more transgender patients	10 (29.4)	5 (27.8)	.90
	VA-wide email announcements about the program	14 (41.2)	9 (50.0)	.54
	Guidance on how to talk with patients about the program	17 (50.0)	8 (44.4)	.70
Number of years working in VA, mean (SD)		7.0 (0.8)	6.8 (1.4)	.45

^a^Not applicable.

^b^VA: Department of Veterans Affairs

### Quantitative Evaluation

#### Overall Analysis

Comparison of the three demographic characteristics between the census of providers who had encountered a transgender patient during the study period and the random sample of survey participants showed no significant differences in facilities (*P*=.36), number of visits (*P*=.48), and number of patients (*P*=.31) (data not shown). Of the 279 eligible providers, 53 responded to the survey (19% response rate). Providers were evenly distributed across disciplines (Table 3).

Among primary care practitioners, 5 were physicians, 3 were nurse practitioners, and 10 were “other” (ie, nurse practitioner in gynecology, art therapist, blind rehabilitation, clinical pharmacy specialist, physician in physical medicine and rehabilitation, nonspecific primary care provider, certified nutrition specialist, psychiatric nurse practitioner, registered dietitian nutritionist, vascular surgeon, or inpatient physician). On an average, the practitioners worked in the VA for 6.9 years (SD 5.3; range, ≤1-25 years). A total of 35 respondents (66%) viewed their VA facility as urban; 15 (28%), as rural; and 3 (6%), as highly rural.

Over half (53%) of the sample had no training in providing transgender health care before working in the VA. Among those who had prior training, 11% had 1 hour, 21% had >1 to <3 hours, and 15% had >3 hours of training. In addition, half of the sample (50%) indicated that while they were working for the VA, they received some form of training on providing care for transgender patients.

Nearly one-third (32%) of the providers encountered only 1 transgender patient in the previous 12 months, and 5 providers (9%) did not encounter any transgender patients in the last 12 months. The majority of the providers (87%) had encountered 1-9 transgender patients over the last 12 months, and 2 providers encountered >10 transgender patients.

**Figure 1:**
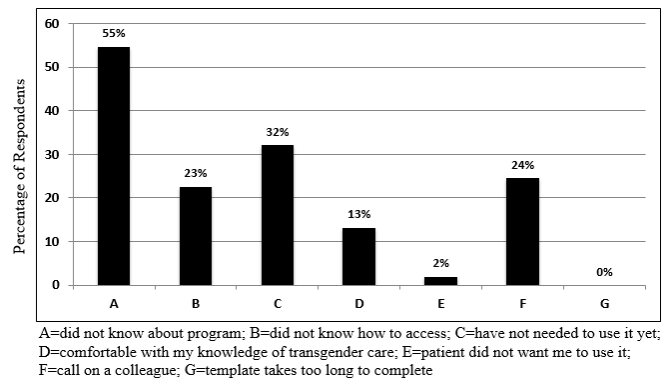
Reasons for not using e-consultation from 53 provider surveys.

**Figure 2:**
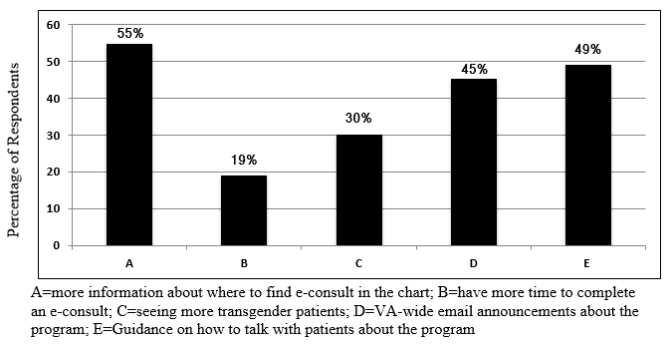
How to improve use of e-consultation from 53 provider surveys. VA: Department of Veterans Affairs.

The most-common reasons for nonuse of the e-consult were as follows: no knowledge about it, no need for it, and receipt of help from a colleague (Figure 1). The most-common suggestions to improve the program use were provision of more information about how to find and access e-consult through the EHR, VA-wide email announcements about the program, and guidance on how to talk with patients about the program (Figure 2).

#### Urban and Rural Differences

Post hoc exploratory analyses were conducted on the basis of the self-reported locale of the providers. Rural and highly rural respondents were combined in one category because of the small number of providers in highly rural facilities. We found no overall differences between urban and rural providers (Table 3).

## Discussion

### Principal Findings

Increase of provider knowledge about transgender patients’ needs can reduce misconceptions and stigma and facilitate high-quality care provision [1,15]. The VA has improved transgender health care by becoming the first and largest US health care system to design and implement a national e-consult program for transgender health care to “respond to specific inquiries on a case-by-case basis” [10].

Because formal transgender health care is relatively new to the VA, it is crucial to understand health care providers’ experiences with a program designed to enhance their practice. Lessons learned can guide improvement and inform other health care organizations that may want to develop or replicate such a program. Some large health care organizations have implemented e-consult programs for several system needs, mainly those facilitating low-cost connections with specialists and continuity of care within the system [16-18]. The implementation of e-consults as a relatively inexpensive, structural service that consolidates clinical expertise into an accessible resource can be an asset for vulnerable populations and providers who serve them. Our study revealed the abovementioned assets among providers caring for patients who are transgender (eg, increased provider comfort with and confidence in transgender care provision) and elucidated the importance of the program in ensuring flexibility in response to provider’s dynamic needs (eg, structured responses to reduce response time).

Because the VA operates the single-largest health care system in the United States, we were able to specifically examine the experiences of providers at rural sites. Qualitative data showed that rural providers who have used e-consult faced unique challenges in providing quality care to transgender veterans. In particular, they did not have on-site providers with knowledge of and experience in transgender-specific care, who they could consult about the veteran. At times, they were unable to follow the e-consult recommendations they received for a specific patient, because the recommendations were not feasible at their location. Thus, the e-consult program did not provide local resources needed to follow providers’ advice. These themes highlight a conundrum that required more directed inquiry, such as exploring how providers in rural areas can deliver the same quality of care as their urban peers, consolidating e-consult responses to rural providers in order to develop tailored guidelines for providing transgender care in underresourced areas, or convening system-focused consensus meetings to bring together providers and administrators from both urban and rural settings to discuss creative quality-improvement strategies.

Although our quantitative analyses showed no statistically significant differentiation between urban and rural providers, the results suggested that rural respondents may be less likely than their urban peers to indicate that they have colleagues who can be informally called for help, reiterating that rural areas generally have limitations to health care infrastructure and availability of providers [19-21], which could have negative effects for care of unique minority populations such as transgender individuals [22-24]. Further research is needed to develop a better understanding of transgender health from the inputs of both providers and patients, specifically in rural settings.

Our results are consistent with existing research findings that highlight ongoing barriers to provision of quality transgender- specific care in VA and nonVA settings [25-28]. Although continually revised standards of care for transgender patients have been available through the World Professional Association for Transgender Health for over 2 decades [29], the majority of health care professionals do not receive training in transgender health [1]. For instance, in a survey of 132 deans representing medical schools in the United States and Canada, one-third reported 0 hours of LGBT-related content delivered during clinical training years and only 30% reported inclusion of gender transition in the required curriculum [30]. In a sample of nearly 250 third- and fourth-year medical students, only 56% chose the correct definition of the term “transgender” [31]. Unsurprisingly, some of the frequently cited barriers to accessing care among transgender patients are the lack of knowledgeable providers (regarding body, identity, expression, etc, for transgender patients and transgender-specific care) [15,32,33] or the need for the patient to educate the provider [4], which may be magnified for patients in rural and remote locations [22,34]. Although medical training and curriculum need to include transgender topics [35], health systems face a concomitant, immediate need to equip current providers with necessary resources in order to provide transgender-specific health care for transgender patients.

### Limitations

We noted several limitations to our study. The qualitative analyses relied on self-reported data from interviews with providers who used the VA transgender e-consult program, which may not replicate direct observational data. Although our relatively small and homogeneous sample was suitable for qualitative analysis, we may not have captured the full range of provider experiences with the VA transgender e-consult program. Notably, the majority of the sample had only used the e-consult program once and one person had used it 7 times; perceptions of the program could vary based on familiarity. In addition, due to the qualitative nature of the study, our results may not be representative or generalizable; this limitation should be considered when interpreting the results before applying our conclusions to other providers and settings.

For the quantitative survey, we identified providers who encountered transgender patients (ie, the use of ICD-9 and ICD-10 diagnosis codes), which is a proxy method of identifying transgender patients; therefore, the sampling may have resulted in a conservative estimate of providers. The quantitative response rate was low, limiting the statistical power of analyses and generalizability of findings. Moreover, because we did not have data on the disciplines for the 14,502 providers in the pool that was sampled, we could not ascertain if providers from certain disciplines were more inclined to respond to the survey, which may have introduced response bias. Future studies with more-frequent and varied follow-up strategies to increase the response rate could alleviate issues of generalizability and response bias. Furthermore, as a pilot study, the survey was limited in scope and did not collect additional information about providers (eg, sex, race or ethnicity, and granular data regarding specialty training).

### Conclusions

This mixed-methods study offers insight into provider experiences with the VA transgender e-consult program and conveys lessons of implementation that can guide other health care organizations to create similar programs (eg, better and more-frequent advertising of the program, infographics or examples about how to initiate an e-consult, or structured advice for providers to talk with patients about e-consults). Our results suggest that the program is useful for providers, but there are several barriers to its use among providers. Additionally, rural providers face unique challenges in care provision for transgender patients, including the lack of access to necessary resources.

Future studies with a larger, more-diverse sample of urban and rural health care providers as well transgender veterans who were involved in the e-consult program (patients must consent for their providers to submit an e-consult) are warranted. The present study included only the providers who asked for e-consults and not the providers who answered the e-consults. Further research, probably via a dyadic approach, could better investigate the entire course of e-consult use. Additionally, a global study of VA e-consults would allow comparisons across consult content (eg, diabetes, dermatology-related, and transgender care) to discern challenges that may be universal to the e-consult format in contrast to challenges that may be unique based on the health care need. Examining transgender e-consults from the patient’s perspective would allow the health system and its providers to learn how consumer experience (eg, patient-provider interactions, patient outcomes, or satisfaction) may be influenced by the use of e-consults [36].

In summary, an e-consult is a relatively low-cost system improvement that health care organizations can implement to support providers in caring for transgender patients. As examples of best-practice suggestions for transgender health accumulate [37-39], feasible, in-house resources will be key for their replication and adaptation across health care organizations.

## Abbreviations

e-consult: e-consultation
EHR: electronic health record
ICD: International Classification of Diseases
LGBT: lesbian, gay, bisexual, and transgender
VA: Department of Veterans Affairs
